# Primary and secondary bacteremia caused by mdr bacteria in ICU patients

**DOI:** 10.1186/2197-425X-3-S1-A885

**Published:** 2015-10-01

**Authors:** E Pappa, G Sarris, H Pavlou, M Eforakopoulou

**Affiliations:** ICU, KAT General Hospital, Athens, Greece

## Introduction

Bacteremia caused by multidrug-resistant bacteria brings about a very serious problem and increases the patients' mortality in ICU.

## Objectives

The aim of this study is to look into the incidence and clinical characteristics of the primary and secondary bacteremia due to multidrug-resistant bacteria in ICU patients.

## Methods

For a 3 months period we have studied all cases of primary and secondary bacteremia occurred in patients hospitalized in ICU of KAT Hospital in Athens. The patients' demographic data, the reason of admission, the day when bacteremia occurred, the cultured microbe and its resistance to antibiotics as well as the duration of patients' hospitalization in ICU have been recorded. The statistical significance was resulted from the t-student and chi-square tests.

## Results

58 cases of bacteremia were recorded in 27 ICU patients. 46 of them (79%) were primary and 12 (21%) were secondary bacteremia. Multidrug-resistant strains have been developed in 26 % of the primary and in 58% of the secondary bacteremia (p < 0.05). Klebsiella has been the most frequent germ in secondary bacteremia (p < 0.0001) and is associated with the long term hospitalization in ICU (p < 0.05). Bacteremia caused by multidrug-resistant strains had no correlation with the age and the reason of admission but it was significantly associated with the duration of hospitalization in ICU (p < 0.01).

## Conclusions

Multidrug-resistant bacteria are more often in secondary bacteremia. The length of ICU stay is important risk factor for bacteremia caused by multiresistant strains.
